# Migration and Transformation of Arsenic in Rice and Soil under Different Nitrogen Sources in Polymetallic Sulfide Mining Areas

**DOI:** 10.3390/life12101541

**Published:** 2022-10-04

**Authors:** Shuhua Yao, Dan Yang, Xuexia Zhang, Lei Shi, Xiaoxia Zhang

**Affiliations:** 1 Liaoning Engineering Research Center for Treatment and Recycling of Industrially Discharged Heavy Metals, Shenyang University of Chemical Technology, Shenyang 110142, China; 2 National-Regional Joint Engineering Research Center for Soil Pollution Control and Remediation in South China, Guangdong Key Laboratory of Integrated Agro-Environmental Pollution Control and Management, Institute of Eco-Environmental and Soil Sciences, Guangdong Academy of Sciences, Guangzhou 510650, China

**Keywords:** mineral nutrients, heavy metal, acid mine drainage, polluted paddy soil

## Abstract

Nitrogen (N) fertilizer affects the migration and transformation of arsenic (As) in soil and rice. We conducted pot experiments and studied the effects of 0.1, 0.2, and 0.4 g∙kg^−1^ N levels of NH_4_Cl, (NH_4_)_2_SO_4_, and NH_4_NO_3_ fertilizers on the As bioavailability in the As-contaminated inter-rhizosphere soil and As accumulation in the rice organs. The results showed that the concentration of bioavailable As in the rice rhizosphere soil was significantly negatively correlated with pH under the 0.4 g∙kg^−1^ N level of each fertilizer. At the same N level, while the As concentration was maturity stage > tillering stage in rice stems and leaves treated with NH_4_Cl and (NH_4_)_2_SO_4_, it was the opposite in roots. This suggests that the transfer of As from roots to stems and leaves mainly occurs in the late stage of rice growth under the condition of only NH_4_^+^-N fertilizer applying. The As concentration in rice aboveground organ (grains and stems–leaves) decreased with the increasing N application under the same N fertilizer treatment condition during the mature stage. In addition, the As concentration in rice grains treated with (NH_4_)_2_SO_4_ was the lowest. This result indicated that SO_4_^2−^ and NH_4_^+^-N had a significant synergistic inhibition on the As accumulation in rice grains. It was concluded that appropriate (NH_4_)_2_SO_4_ levels for As-contaminated paddy soils with high sulfur (S) contents would obtain rice grains with inorganic As concentrations below 0.2 mg·kg^−1^.

## 1. Introduction

The mining industry can cause significant damage to terrestrial and aquatic environments at certain scales, and the mining process generates large amounts of waste that is deposited in terrestrial or aquatic systems. This results in the heavy metal pollution of air, soil, river water, and groundwater [1]. One of the most common forms of pollution near sulfide mines is the oxidation of weathered S-containing minerals that releases heavy metals and acidic wastewater, and these are then released into surface water, soil, and groundwater [2,3,4]. This leads to excessive levels of heavy metals in crops, such as rice downstream of agricultural fields [5], seriously damaging plant growth and human health [6]. High concentrations of sulfate ions in mining wastewater can lead to soil acidification and an excessive soil S content, and this may develop into acidic sulfate soils that contain many toxic substances. This leads to soil degradation and even damage to crop growth [7,8,9]. As a toxic heavy metal, the prolonged presence and accumulation of As in soil can affect soil physicochemical properties and crop growth and even have toxic effects through the food chain, affecting the health of organisms [10]. Plant nutrient regulation is an important tool to mitigate and manage agricultural soils, and mineral nutrients can effectively control the uptake and accumulation of heavy metal elements by crops [11]. Nitrate (NO_3_^−^) and ammonium (NH_4_^+^) salts as N fertilizers are the primary ways to obtain N for non-legume crops such as rice, but As can reduce the N fertilizer transportation and interfere with the availability of inorganic N [12]. Uddin et al. [13] studied the relationship between the application of nitrogen fertilizer and the release of arsenic from sediments in the polluted area and found that the application of N fertilizer would significantly promote the release of As from sediments in the polluted area of Bangladesh to groundwater. The study of Liu et al. showed that the urea application increased the exchangeable As concentration in the soil [14]. It has been shown that S has an inhibitory effect on the bioavailability and transport of As [15] that significantly reduces the toxicity of As to plants and promotes plant growth [15,16]. Manju et al. found that plant seedlings were affected by As stress, and that was because reduced glutathione and cysteine-containing S had a protective effect on them by reducing the uptake of As in plants [17]. This result was the same as that of Tuli et al. [18]. By studying the effect of S on black algae subjected to As stress, Srivastava et al. found that S increased plant tolerance to As by modulating plant antioxidant mechanisms and increasing the enzymatic activity of the antioxidant system [19]. It has been reported that S and N interact in plants. For example, S addition reduces the NO_3_^−^ content and increases N accumulation in plants [20]. However, there are few reports about the effects of S and N used together on As toxicity [15]. Therefore, in this study, we investigated the bioavailability of As in plant inter-rhizosphere soil and the uptake of As in different rice organs by comparing different N sources and N levels. We accomplished this by studying rice growth in a polymetallic S mining area and conducting pot experiments to explore the appropriate N fertilizer species and application levels considering the combined effects of S and N.

## 2. Materials and Methods

### 2.1. Test Soil

This experiment studied a heavy-metal-contaminated paddy field with more than 50 years of irrigation history (since the 1970s) that was irrigated with acidic mine wastewater from the Dabaoshan polymetallic sulfide mining area. A determination of the heavy-metal concentrations in the soil of this paddy field found elements such as As, lead (Pb), and Cd. The concentrations were less than the risk control value for soil contamination of agricultural land, but they were greater than the risk screening value. In addition, edible agricultural products, such as rice, may not meet the requirements of the national standards for food safety. In this study, soil samples from 0 to 20 cm of the cultivated layer were used as the test soil, air-dried, sieved (2 mm), and mixed well for use. The physical and chemical properties of the test soils are shown in Table 1.

### 2.2. Pot Experiment

The effect of N fertilizer application on the soil–rice transport of As was investigated by using Choukoukoku (Oryza sativa L. cv.) with a high Cd accumulation. The rice grains were sterilized by using NaClO at a concentration of 0.5% for 20 min, rinsed several times, and then soaked in deionized water. They were removed after one day and placed in moist gauze for cultivation and cultivated until the seeds germinated to approximately 1 cm in height. They were then removed and placed in pots that contained quartz sand and deionized water for further cultivation. Two plants per pot were utilized for the soil-cultivation experiment. The experiment was conducted in ordinary plastic bottles with a diameter and height of 20 cm. Two days prior to transplanting, 3.8 kg of the soil was placed into the pots, and 17.6 g of KH_2_PO_4_ with different contents of N fertilizer was weighed, mixed, and dissolved in 1 L of water. This was poured into the pots after complete dissolution. The plants were watered in the morning and evening.

Nine different fertilization treatments according to different N fertilizers and N levels were administered to the rice, as shown in Table 2, and there were five replicated pots (ten plants) for each treatment.

### 2.3. Rice Treatments

All rice seedlings were transplanted on 21 May 2012, and two pots of rice plants were collected as tillering samples on 2 July. Three pots of rice plants were harvested as maturity samples after the grains matured and were treated with a three-day roasting. However, the maturity stage of the rice differed among treatments, and the rice samples were harvested on 23 August for the 0.1 and 0.2 g∙kg^−1^ N levels of the three forms of N fertilizers treatments and 0.4 N level of NH_4_NO_3_ treatments; on 28 August to 6 September for the 0.4 N level of (NH_4_)_2_SO_4_ treatment; and 3 September to 14 September for the 0.4 N level of NH_4_Cl treatment. Days of the whole rice growth period under treatment conditions with different N forms and levels are shown in Table 3. Photos of the rice under treatment conditions with different N forms and levels were taken on 23 August 2012. They are provided in Supplementary Figure S1.

Each rice plant was treated as a replication. The rice samples were cut from the roots at harvest, and the stems–leaves and the rice grains were collected. The roots were then slowly removed, the debris was removed, and the roots and inter-rhizosphere soil were collected as research samples. The inter-rhizosphere soil was air-dried, and the debris was removed; the soil was then ground, passed through 10- and 100-mesh sieves, and stored in separate polyethylene plastic bags for use. The obtained roots, stems–leaves, and rice grains were rinsed with tap water and deionized water successively, green-killed at 105 °C for 0.5 h, dried at 60 °C to a constant weight, ground, passed through a 60-mesh sieve, and then stored for use.

### 2.4. Sample Testing and Data Analysis

The pH was determined by using the glass electrode method (water–soil ratio of 1:2.5). We digested the soil with HNO_3_-HClO_4_-HF solutions and determined the heavy-metal and S concentrations in the soil by using an inductively coupled plasma–optical emission spectrometer (ICP–OES, ICP-5000, Focused Photonics, Inc., Zhejiang, China). The detection limits measured by the ICP–OES were 0.5 µg·L^−1^ for Cd, 1 µg·L^−1^ for Cr, 0.5 µg·L^−1^ for Cu, 0.5 µg·L^−1^ for As, 0.5 µg·L^−1^ for Pb, 0.2 µg·L^−1^ for Zn, 0.5 µg·L^−1^ for Fe, and 0.2 mg·L^−1^ for S. The bioavailable concentration of S in the soil was leached by using the 1:5 suspensions of soil and 0.008 mol∙L^−1^ Ca(H_2_PO_4_)_2_–2 mol∙L^−1^ CH_3_COOH mixed solution at 180 revolutions per minute (rpm) for 1 h, and then it was determined by using the ICP–OES. The bioavailable concentration of As in the soil was determined by using the AFS-830 atomic fluorescence photometer after being leached using a 1:2 suspension of soil and a mixed solution of 0.005 mol∙L^−1^ DTPA, 0.01 mol∙L^−1^ CaCl_2_, and 0.1 mol∙L^−1^ TEA at 180 rpm for 2 h. The rice organ samples were analyzed by using HNO_3_-HClO_4_ (3:1, *v*:*v*) mixed acid digestion.

The bulk density was traditionally assessed by gravimetric methods. The cation exchange capacity (CEC) was determined by using ammonium acetate [21]. The organic matter content was determined by using the Walkley–Black procedure [22]. For total phosphorus (P), soils were digested with concentrated HClO_4_–H_2_SO_4_. Extracted P was measured by using the molybdenum antimony blue colorimetry method [23]. Alkaline hydrolyzed N was determined by a micro-diffusion technique after alkaline hydrolysis. Soil total N was determined by the semi-micro Kjeldahl digestion procedure [24]. Total potassium (K) was determined by digesting in sodium hydroxide and then measured by atomic absorption spectrometer. The reagents used in the experimental pretreatment process were guaranteed reagents (GRs).

One standard solution was added for every 20 samples during the samples analysis, and the internal standard solution was used to control the quality and reduce the error. The recoveries of As were higher than 95% for both the soil and plant samples, and the relative standard deviations (RSDs) had a precision of greater than 10%.

All assay data were calculated by using the IBM SPSS Statistics 25 for the mean and standard deviation and expressed in Duncan. Different N level treatments of each fertilizer were compared by using least significant difference at *p* < 0.05. Histograms were plotted by using Origin 2018.

## 3. Results and Discussion

### 3.1. Bioavailable As and pH in Rice Inter-Rhizosphere Soil under Different N Fertilizer Treatment Conditions

**Figure 1:**
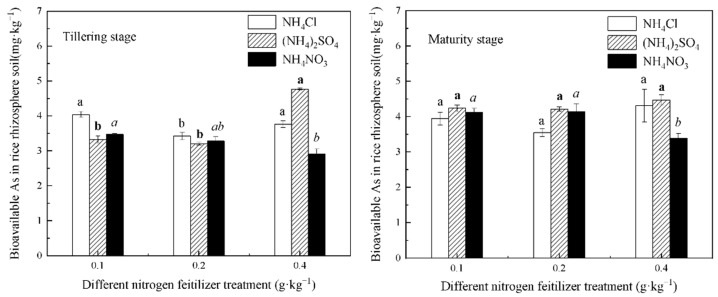
Concentration of bioavailable As in the rhizosphere soil under the different forms and levels of N fertilizer treatment conditions. Different letters in figure mean that there are significant differences among the N levels of each fertilizer (*p* < 0.05).

The effects of different N fertilizer treatment conditions on the bioavailable As concentration in the inter-rhizosphere soil of the rice during the tillering and maturity stages are shown in Figure 1. During the tillering stage, the bioavailable As concentration under 0.1 g∙kg^−1^ and 0.2 g∙kg^−1^ N fertilizer treatment conditions was NH_4_Cl > NH_4_NO_3_ > (NH_4_)_2_SO_4_. During the maturity stage, the bioavailable As concentration under 0.1 g∙kg^−1^ and 0.2 g∙kg^−1^ N fertilizer treatment conditions was (NH_4_)_2_SO_4_ > NH_4_NO_3_ > NH_4_Cl. The bioavailable As concentration at both periods under 0.4 g∙kg^−1^ N fertilizer treatment condition was (NH_4_)_2_SO_4_ > NH_4_Cl > NH_4_NO_3_. These results indicated that, during the different periods, the different N fertilizer treatment conditions had different effects on the bioavailable As in the inter-rhizosphere soil. During the tillering stage, the bioavailable As concentration in the soil under the same N application was NH_4_Cl > NH_4_NO_3_. At the 0.4 g∙kg^−1^ N level, the bioavailable As concentration in the soil treated by NH_4_NO_3_ (NH_4_^+^-N coexisted with NO_3_^−^-N) was significantly lower than that treated by NH_4_Cl and (NH_4_)_2_SO_4_ (NH_4_^+^-N coexisted alone); this indicated that NO_3_^−^-N could significantly decrease the As bioavailability under a high-N-level-fertilizer treatment condition.

We compared the As concentration changes during the tillering stage and the maturity stage in Figure 1, and we found that the change trends of the bioavailable As concentration in the inter-rhizosphere soil treated with the same N fertilizer were similar with the increasing N level during the both stages. For the NH_4_Cl treatments, the As concentration first decreased and then increased. For the (NH_4_)_2_SO_4_ treatments, the As concentration first remained nearly unchanged, and then it increased. Moreover, for the NH_4_NO_3_ treatments, the As concentration first remained nearly unchanged, and then it decreased. Figure 1 showed that the bioavailable As concentration in the rice rhizosphere soil decreased slightly with the increase of the NH_4_NO_3_ level. The reason might be that the addition of NO_3_^−^ to the inter-rhizosphere soil may inhibit the reduction of Fe^3+^ or promote the oxidation of Fe^2+^, which, in turn, leads to the adsorption or co-precipitation of As with Fe^3+^ minerals [25]. Thus, the bioavailable As in the soil decreased.

Many studies have found that N fertilizers can affect the pH of plant soils [26,27]. For example, Liu et al. studied the relationship between urea application and soil exchangeable As concentration, and the results showed that the urea application increased the pH of the soil and increased the exchangeable As concentration in the soil [14]. In contrast, the bioavailable As concentration in the soil can be affected by pH due to the alkaline environment, and a large amount of OH^−^ can compete with arsenate anion for sorption sites. This results in a decrease in the As sorption. However, the phenomenon is opposite for an acidic environment where the bioavailable As concentration increases with the As sorption increases [28]. In this study, under the same N level (except 0.4 g∙kg^−1^), the bioavailable As concentration in the rice rhizosphere soil during tillering stage was NH_4_Cl treatment > NH_4_NO_3_ treatment > (NH_4_)_2_SO_4_ treatment, but the phenomenon was opposite during the mature stage: (NH_4_)_2_SO_4_ treatment > NH_4_NO_3_ treatment > NH_4_Cl treatment. Therefore, it is necessary to apply different varieties of N fertilizers for the different fertility stages of rice to control the As concentration in rice.

**Figure 2:**
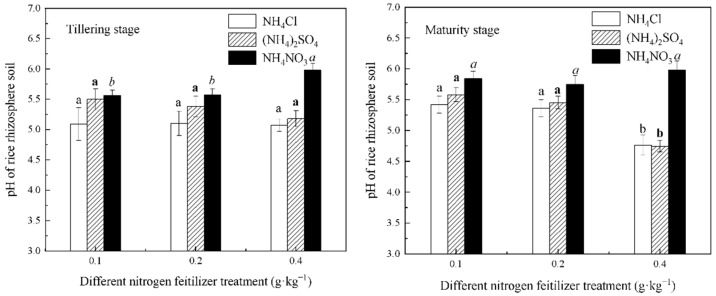
The pH of the rice rhizosphere soil under the different forms and levels of N fertilizer treatment conditions. Different letters in the figure mean that there are significant differences among the N levels of each fertilizer (*p* < 0.05).

Figure 2 shows the effect of the different N fertilizers and different N applications on the pH of the inter-rhizosphere soil of rice during the tillering and maturity stages. The inter-rhizosphere soil pH of the NH_4_NO_3_ treatment was greater than that of the NH_4_Cl and (NH_4_)_2_SO_4_ treatments. The soil pH of the NH_4_Cl and (NH_4_)_2_SO_4_ treatments gradually decreased with the increasing N application, while the NH_4_NO_3_ treatment showed the opposite trend. The concentration of bioavailable As in the rice rhizosphere soil was significantly negatively correlated with the pH under the 0.4 g∙kg^−1^ N level of each fertilizer.

After entering the soil, N fertilizers first change the soil pH and thus affect the activity of the soil’s heavy metals, and different forms of N fertilizers have different effects on the inter-rhizosphere soil’s pH [29,30,31]. Md. Shamim Uddin et al. studied the effect of N fertilizers on As release from groundwater and found that the N concentration was positively correlated with the As concentration, and NH_4_^+^-N significantly promoted As uptake compared to NO_3_^−^-N [13]. This happened because, when the plant roots absorb NH_4_^+^-N, they secrete H^+^, and when the plant roots absorb NO_3_^−^-N, they secrete OH^−^. In addition, the secretion of the two ions changed the pH of the inter-rhizosphere soil. Hence, the two N fertilizers had opposite effects on the uptake of As. Under the condition of the same N level, NH_4_NO_3_ contains more NO_3_^−^-N than both NH_4_Cl and (NH_4_)_2_SO_4_; hence, the soil treated with NH_4_NO_3_ was acidified less, and the pH was higher than it was for the other two N fertilizers. Therefore, the pH of the rice inter-rhizosphere soil treated with NH_4_NO_3_ (shown in Figure 2) was higher and increased with the increasing NH_4_NO_3_ dose. SO_4_^2−^ also had an effect on the inter-rhizosphere soil’s pH and bioavailable As concentration, besides NO_3_^−^ and NH_4_^+^. As shown in Figure 2, all pH values were (NH_4_)_2_SO_4_ treatment > NH_4_Cl treatment, except for the similar pH values under the 0.4 g∙kg^−1^ N level of (NH_4_)_2_SO_4_ treatment and NH_4_Cl treatment during the maturity stage. This indicated that SO_4_^2−^ had an inhibitory effect on the pH decreasing caused by NH_4_^+^. Chen et al. studied the effect of SO_4_^2−^ on the As adsorption by river sand. They found that As existed in various forms in the inter-rhizosphere soil, and As(V) adsorption was reduced by 35.87% compared with the treatment without the addition of SO_4_^2−^ [32]. Their experiment proved that SO_4_^2−^ could inhibit the adsorption of As in the soil [32], and this was the same as the results of this study at the early stage of rice growth with the N levels of 0.1 and 0.2 g∙kg^−1^. This may have been due to an increase in the soil pH caused by SO_4_^2−^, along with ion exchange with As (V), resulting in a decrease in the As adsorption [32]. However, the bioavailable As concentration in the soil treated with the (NH_4_)_2_SO_4_ treatment was greatest at the 0.4 g∙kg^−1^ N level at both growth stages. Moreover, it was greatest under the 0.1 and 0.2 g∙kg^−1^ N levels at maturity with the increasing time. This indicated that SO_4_^2−^ promoted As bioavailability under the condition of a high N level or long time. In the early stage of this experiment, we also studied the soil of the sulfide mining area, and the amount of total S in the experimental soil was 443.66 mg∙kg^−1^. However, the bioavailable S concentration was 133.38 mg∙kg^−1^, and the bioavailable S concentration was 6.2 times higher than the average bioavailable S value of Guangdong Province. These data indicated that the study soil had a large amount of SO_4_^2−^, and these SO_4_^2−^ ions were highly migratory and active. This significantly increased the bioavailable S concentration. Figure 1 showed that the bioavailable As concentration at the maturity stage was higher than at tillering stage under the condition of the same NH_4_NO_3_ dose treatment. The result indicated that the bioavailable As concentration in the rice rhizosphere soil increased with the increasing growth time. This result may be caused by the bioavailable S in the soil. The bioavailable S in the soil may have activated As under the condition of long rice growth time and NH_4_NO_3_ treatment. Therefore, it is worth paying attention to the influence of high S environment on As in soil.

### 3.2. As Concentration in the Rice Roots under Different N Fertilizer Treatment Conditions

**Figure 3:**
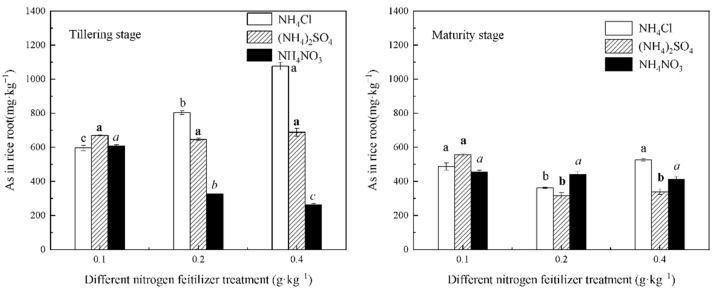
As concentration in the rice roots under the different forms and levels of N fertilizer treatment conditions. Different letters in figure mean that there are significant differences among the N levels of each fertilizer (*p* < 0.05).

Figure 3 shows the effects of the different treatments of N fertilizer and N levels on the As concentration in the rice roots. It can be seen that different fertilizers had different effects on the As concentration in rice roots under the same conditions of N application during the same fertility period. During the tillering stage, the As concentration in the rice roots increased from 596.09 mg∙kg^−1^ to 1076.92 mg∙kg^−1^ with the increasing application level of NH_4_Cl, indicating that NH_4_Cl promoted As uptake. The As uptake by the rice roots decreased with an increase in the NH_4_NO_3_ application, and from 607.21 mg∙kg^−1^ to 261.42 mg∙kg^−1^, there was a decrease of 56.95%. The differences in the effects of the two N fertilizers on the As accumulation indicated that NO_3_^−^ had a significant inhibitory effect on the As concentration in the rice roots during the early stages of plant growth. (NH_4_)_2_SO_4_ had no significant effect on the As accumulation, and this may be due to the balance between the promoting effect of NH_4_^+^ and the inhibiting effect of SO_4_^2−^ on the As uptake during the tillering stage. In addition, with the growth of plants, SO_4_^2−^ in the soil entered the plant roots, and when its concentration was greater than NH_4_^+^, the inhibitory effect of As was enhanced. Hence, the accumulation of As decreased. In addition, it was also found that the difference in the As accumulation between the three N fertilizers at 0.1 g∙kg^−1^ of N application during the tillering stage was small, indicating that low N levels and N fertilizer varieties had no significant effect on the As uptake by rice roots during the tillering stage.

The As concentration decreased and then increased when NH_4_Cl and (NH_4_)_2_SO_4_ were applied during the maturity stage, and the accumulation of As in the rice roots was the least at 0.2 g∙kg^−1^ of N application. The inhibitory effect of (NH_4_)_2_SO_4_ on the As accumulation was more obvious. This was likely because SO_4_^2−^ facilitated the formation of iron film on the root surface of the rice and hindered the uptake of As [33]. When NH_4_NO_3_ was applied, the As accumulation was nearly unchanged with an increase in the N application, indicating that NH_4_NO_3_ had a significant inhibitory effect on As uptake during only the early stage of rice growth, while the As concentration during the late stage of rice growth was not affected by NH_4_NO_3_. One study found that the total As concentration in fern increased with an increase in N fertilizer when different N fertilizers were applied, and the As concentration in fern was NH_4_HCO_3_ > (NH_4_)_2_SO_4_ > urea > Ca(NO_3_)_2_ > KNO_3_; there was no significant difference between the NO_3_^−^-N treatment and the control treatment [34]. This result was consistent with the effect of NH_4_NO_3_ on the As accumulation in rice roots at maturity found in this experiment.

Figure 3 showed that the concentration of As in the rice roots during the tillering stage was higher than that during the maturity stage, except for NH_4_NO_3_ treatments at the 0.2 and 0.4 g∙kg^−1^ N levels. This was because the amount of iron film on the root surface during the early stages of rice growth was low, and the ability to accumulate As was strong. In addition, the As content accumulated by the roots was high, and this accelerated the migration of As from the soil to the root surface and increased the As concentration in the rice roots during the tillering stage. With the rice growth, As migrated from the underground part to the aboveground part, leading to a decrease in the As accumulation in the rice roots at maturity [35].

A comparison of Figure 1 and Figure 3 revealed that the different N fertilizers had different effects on the As concentration in the inter-rhizosphere soil and rice roots. The bioavailability of As in the inter-rhizosphere soil of the rice during the tillering stage was NH_4_Cl treatment > NH_4_NO_3_ treatment > (NH_4_)_2_SO_4_ treatment, while the As concentration in the roots was NH_4_Cl treatment > (NH_4_)_2_SO_4_ > NH_4_NO_3_ treatment. This difference might be because NO_3_^−^, as a strong oxidizer, oxidizes As(III) to As(V), and As(V) is easily adsorbed by iron (hydrogen) oxides, thus reducing the As uptake in rice roots [36].

### 3.3. As Concentration in Rice Stems and Leaves under Different N Fertilizer Treatment Conditions

**Figure 4:**
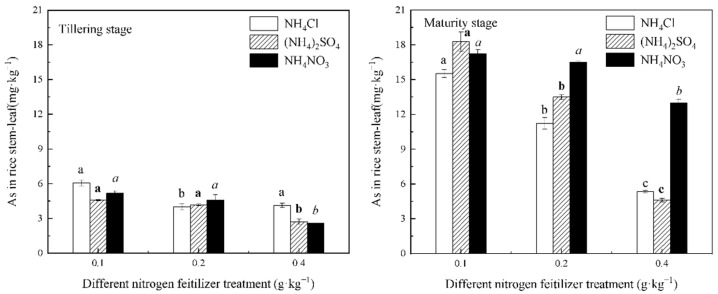
As concentration in the rice stems and leaves under the different forms and levels of N fertilizer treatment conditions. Different letters in figure mean that there are significant differences among the N levels of each fertilizer (*p* < 0.05).

The As concentrations in the stems and leaves of all three N fertilizer treatments, except for the NH_4_Cl treatment at the tillering stage, decreased with the increasing N application in both stages of rice growth, as shown in Figure 4. This indicated that the rice stems and leaves’ accumulation of As was inhibited with an increasing N level. In contrast, the As concentration in rice stems and leaves during the maturity stage was greater than that during the tillering stage at the same N level. This result indicated that As in rice stems and leaves accumulated largely during the rice’s mature stage, while As in rice roots accumulated largely during the rice tillering stage except for NH_4_NO_3_ treatments at 0.2 and 0.4 g∙kg^−1^ of N levels. The higher As concentration in the rice stems and leaves during the later stages of plant growth may have been caused by the increased accumulation of As that translocated from the roots to the stems and leaves with the plants’ growth time.

In addition, Figure 4 shows that the As concentration in the rice stems and leaves was NH_4_NO_3_ treatment > NH_4_Cl treatment during the rice’s maturity stage, indicating that NH_4_NO_3_ is more favorable for As accumulation during the later stages of plant growth compared to NH_4_Cl. This may be because, during the transfer of NO_3_^−^ from the heel to the stem and leaves of the plant, it reaches charge equilibrium with cations, and this facilitates the transfer of some heavy metals to the aboveground portion [37]. In rice roots, however, the As concentration was NH_4_Cl treatment > NH_4_NO_3_ treatment at 0.2 and 0.4 g∙kg^−1^ of N application during the tillering stage, indicating that different N fertilizers have different effects on the As transport in different organs of plants, and the soil pH is not the only criterion for evaluation.

Figure 4 indicated that the As concentration in the rice stem–leaf decreased significantly compared to the rice roots. This result indicated that As was inhibited during the translocation from the rice roots to the stem–leaf, and it has been reported that S-induced thiol ligands can form stable complexes with As and limit the transfer of As to the ground [38,39]. In addition, it can be seen that, under NH_4_Cl treatment conditions and (NH_4_)_2_SO_4_ treatment conditions, while the concentration of As in the rice roots was tillering stage > maturity stage, the concentration of As in the rice stems and leaves was tillering stage < maturity stage. This suggests that the transfer of As from the roots to the stems and leaves mainly occurs in the late stage of rice growth under the condition that only NH_4_^+^-N fertilizer is applied.

### 3.4. As Concentration in Rice Grains under Different N Fertilizer Treatment Conditions

**Figure 5:**
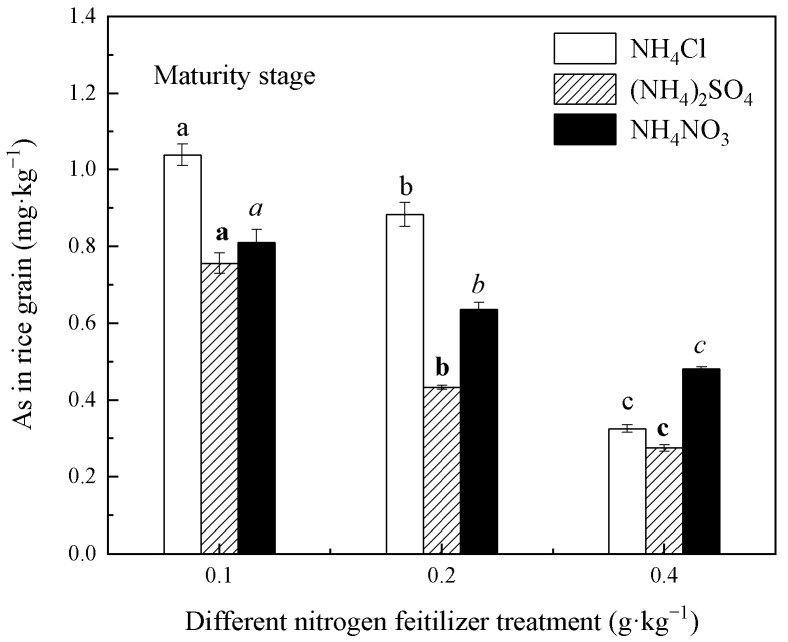
As concentration in rice grains under the different forms and levels of N fertilizer treatment conditions. Different letters in figure mean that there are significant differences among the N levels of each fertilizer (*p* < 0.05).

The effects of three N fertilizers on the As accumulation in rice at different N levels are shown in Figure 5. According to the national food safety standard (GB 2762-2017) [40], the limit standard of inorganic As in rice is 0.2 mg∙kg^−1^. In view of previous measurement experience, the inorganic As concentration accounts for about 1/3 to 2/3 of the total As concentration. According to the results of this experiment, the inorganic As concentration of rice grains might not exceed the standard under the treatment conditions of the 0.2 g∙kg^−1^ N level of (NH_4_)_2_SO_4_ and 0.4 g∙kg^−1^ N level of all three N fertilizers.

With an increase in the N level, the As concentration of rice under the three fertilizers decreased differently. Compared with the 0.1 g∙kg^−1^ N level, the As concentration in rice grains treated with the 0.4 mg∙kg^−1^ N level of NH_4_Cl, (NH_4_)_2_SO_4_, and NH_4_NO_3_ decreased by 68.64%, 63.59%, and 40.29%, respectively. NH_4_Cl and (NH_4_)_2_SO_4_ decreased the As concentration in rice greatly, while the As concentration of rice, except for the 0.4 g∙kg^−1^ N level, was as follows: NH_4_Cl treatment > NH_4_NO_3_ treatment > (NH_4_)_2_SO_4_ treatment. The (NH_4_)_2_SO_4_ treatment group accumulated the least amount of As. In a comparison of Figure 3, Figure 4 and Figure 5, we found that the As concentration was as follows: roots > stems and leaves > grains. The largest As concentration accumulated in the rice roots. If specific measures can be taken to fix it in the roots, this will effectively reduce the transfer of As to the aboveground portions of the rice and lower the As concentration in rice.

Table 3 shows the number of days of rice growth in each experimental group, and it is clear that the 0.4 g·kg^−1^ N levels of NH_4_Cl and (NH_4_)_2_SO_4_ treatments slowed down the growth and maturation of the rice. This may have been related to impaired plant tissue development or function due to high N concentrations, and some studies have shown that the threshold for NH_4_Cl application in the phytoremediation of mycoplasma spinosum was 0.2 g∙kg^−1^ [41]. The N level often used in our experiments is 0.2 g∙kg^−1^ [42].

The effects of N fertilizer on the As accumulation in this experiment were different under the different types, different amounts of N application, and in the different plant organs. During the tillering stage, the NH_4_Cl fertilizer had a promotion effect on the As accumulation in rice roots, stems, and leaves. During the maturity stage, the N fertilizer greatly inhibited the accumulation of As in stems, leaves, and rice grains. The As concentration in the rice’s aboveground organ (grains and stems–leaves) decreased with the increasing N application under the same N fertilizer treatment condition during the mature stage. SO_4_^2−^, NO_3_^−^, and NH_4_^+^ had different effects on the As accumulation, and all the three ions played a restraining role on the As accumulation. In addition, the As concentration in rice grains treated with (NH_4_)_2_SO_4_ was lowest. This result indicated that SO_4_^2−^ and NH_4_^+^-N had a significant synergistic inhibition on the As accumulation in rice grains. Considering the effect of SO_4_^2−^ in the experimental soil, the application of NH_4_^+^-N fertilizer to rice was more beneficial to control the As concentration. Based on the As accumulation in mature rice, it can be concluded that the application of (NH_4_)_2_SO_4_ to rice grown in the acid mine wastewater irrigation area was more favorable to control the As concentration.

In addition, generally the accumulation of As in plants was positively correlated with the availability of As in soil. This study, however, showed that the bioavailable As concentration in soil was positively correlated with the As absorption by rice roots, but not rice grains and rice stems–leaves. Therefore, chemical extraction methods to characterize the bioavailability of As in soil should be improved.

## 4. Conclusions

The study of rice soils in polymetallic sulfide mining areas, using pot experiments, revealed that the application of different N forms and N levels had different effects on the bioavailability of As in the rice inter-rhizosphere soil and the changes in the As concentration in the rice roots, stems–leaves, and rice grain. The concentration of bioavailable As in the rice rhizosphere soil was significantly negatively correlated with pH under the 0.4 g∙kg^−1^ N level of each fertilizer during the same growth period. The high N level makes great changes in the soil pH and bioavailability of As. SO_4_^2−^ promoted As bioavailability under the condition of a high N level or long time. At the same N fertilizer form and level, while the As concentration was at the tillering stage > maturity stage, except NH_4_NO_3_ treatments at 0.2 and 0.4 g∙kg^−1^ of N levels in rice roots, it was tillering stage < maturity stage in rice stems and leaves. This suggests that the transfer of As from the roots to the stems and leaves mainly occurs in the late stage of rice growth under the condition of only NH_4_^+^-N fertilizer applying. The As concentration in rice’s aboveground organ decreased with the increasing N application under the same N fertilizer treatment condition during the mature stage. However, the bioavailable As concentration in soil was positively correlated with the As absorption by rice roots, but not rice grains, stems, and rice leaves. Therefore, chemical extraction methods to characterize the bioavailability of As in soil should be improved. In addition, the As concentration in rice grains treated by (NH_4_)_2_SO_4_ was lowest at the same N level. This result indicated that S and NH_4_^+^-N synergistically reduced As accumulation in rice grains. For the As contaminated soil, the application of an appropriate amount of (NH_4_)_2_SO_4_ will help rice grains accumulate the least amount of As. Thus, this study suggested that (NH_4_)_2_SO_4_ would be a good N source to reduce As phytoextraction for paddy soils with high levels of S concentration and likely would obtain rice grains with inorganic As concentrations below the food safety standards of 0.2 mg·kg^−1^.

## Figures and Tables

**Table 1:** Basic physical and chemical properties of the tested soil.

	Risk Screening Value ^a^ (pH ≤ 5.5)	Risk Intervention Value ^a^ (pH ≤ 5.5)	Test Value
pH			4.21
Cd (mg·kg^−1^)	0.3	1.5	0.54
As (mg·kg^−1^)	30	200	70.28
Cu (mg·kg^−1^)	50		298.25
Zn (mg·kg^−1^)	200		302.66
Cr (mg·kg^−1^)	250	800	46.28
Pb (mg·kg^−1^)	80	400	201.00
Fe (g·kg^−1^)			30.37
S (mg·kg^−1^)			443.66
Bioavailable S (mg·kg^−1^)			133.38
Bulk density (g·cm^−3^)			1.20
organic matter (g·kg^−1^)			11.20
CEC (cmol·kg^−1^)			9.52
Hydrolyzed N (mg·kg^−1^)			10.59
N (g·kg^−1^)			1.10
P (g·kg^−1^)			1.20
K_2_O (g·kg^−1^)			24.2

^a^ Risk screening values and risk intervention values for soil contamination of agricultural land, soil environmental quality national standard of the People’s Republic of China, and risk control standard for soil contamination of agricultural land (GB 15618-2018).

**Table 2:** The application dose of each N fertilizer under the different N-level treatments.

Fertilizer	N (g N·kg^−1^ Soil)		
	0.1 g·kg^−1^	0.2 g·kg^−1^	0.4 g·kg^−1^
NH_4_Cl	7.6 g	15.2 g	30.4 g
(NH_4_)_2_SO_4_	9.4 g	18.8 g	37.6 g
NH_4_NO_3_	5.8 g	11.6 g	23.2 g

**Table 3:** Days of the whole rice-growth period under treatment conditions with different N forms and levels.

Fertilizer	N (g N·kg^−1^ Soil)		
	0.1 g·kg^−1^	0.2 g·kg^−1^	0.4 g·kg^−1^
NH_4_Cl	109 days	109 days	(120–131) days
(NH_4_)_2_SO_4_	109 days	109 days	(114–123) days
NH_4_NO_3_	109 days	109 days	109 days

## Data Availability

The raw data supporting the conclusions of this manuscript will be made available by the authors, without undue reservation, to any qualified researcher.

## Supplementary Materials

The following are available online at https://www.mdpi.com/article/10.3390/life12101541/s1. Figure S1: Photos of rice under treatment conditions with different N forms and levels were taken on 23 August 2012.

## References

[1] Wu Y.G., Xu Y.N., Zhang J.H., Hu S.H. (2010). Evaluation of ecological risk and primary empirical research on heavy metals in polluted soil over Xiaoqinling gold mining region, Shaanxi, China. Trans. Nonferrous Met. Soc. China.

[2] Tiwary R. (2001). Environmental impact of coal mining on water regime and its management. Water Air Soil Pollut..

[3] Navarro M.C., Pérez Sirvent C., Martínez Sánchez M.J., Vidal J., Tovar P.J., Bech J. (2008). Abandoned mine sites as a source of contamination by heavy metals: A case study in a semi-arid zone. J. Geochem. Explor..

[4] Naidu G., Ryu S., Thiruvenkatachari R., Choi Y., Jeong S., Vigneswaran S. (2019). A critical review on remediation, reuse, and resource recovery from acid mine drainage. Environ. Pollut..

[5] Lei M., Tie B.Q., Song Z.G., Liao B.H., Lepo J.E., Huang Y.Z. (2015). Heavy metal pollution and potential health risk assessment of white rice around mine areas in Hunan Province, China. Food Secur..

[6] Zhao F.J., Wang P. (2019). Arsenic and cadmium accumulation in rice and mitigation strategies. Plant. Soil..

[7] Chen M.Q., Wu F.J. (2012). Mechanisms and Remediation Technologies of Sulfate Removal from Acid Mine Drainage. Adv. Mater. Res..

[8] Liu Y., Lin C., Ma Y., Lu W., Wu Y., Huang S., Zhu L., Li J., Chen A. (2009). Toxic effects of two acid sulfate soils from the Dabaoshan Mine on Corymbia citriodora var.variegata and Daphnia carinata. J. Hazard. Mater..

[9] Sokolova T.A., Alekseeva S.A. (2008). Adsorption of sulfate ions by soils (A Review). Eurasian Soil Sci..

[10] Garg N., Singla P. (2011). Arsenic toxicity in crop plants: Physiological effects and tolerance mechanisms. Environ. Chem. Lett..

[11] Zhang X.X., Zhang X.X., Lv S.J., Shi L., Wang R.P. (2020). Migration and transformation of cadmium in rice-soil under different nitrogen sources in polymetallic sulfide mining areas. Sci. Rep..

[12] Finnegan P.M., Chen W. (2012). Arsenic toxicity: The effects on plant metabolism. Front. Physiol..

[13] Uddin M.S., Kurosawa K. (2011). Effect of chemical nitrogen fertilizer application on the release of arsenic from sediment to groundwater in Bangladesh. Procedia Environ. Sci..

[14] Liu X.Y., Zeng Q.R., Zhou X.H., Jiang Z.H., Liao B.H. (2008). The Short-term Changes of Soil pH and Available as by Fertilizing Urea in Contaminated Soils. Chin. J. Soil Sci..

[15] Tang X.J., Li L.Y., Wu C., Khan M.I., Manzoo M., Zou L., Shi J.Y. (2020). The response of arsenic bioavailability and microbial community in paddy soil with the application of sulfur fertilizers. Environ. Pollut..

[16] Dixit G., Singh A.P., Kumar A., Dwivedi S., Deeba F., Kumar S., Suman S., Adhikari B., Shukla Y., Trivedi P.K. (2015). Sulfur alleviates arsenic toxicity by reducing its accumulation and modulating proteome, amino acids and thiol metabolism in rice leaves. Sci. Rep..

[17] Shri M., Kumar S., Chakrabarty D., Trivedi P.K., Mallick S., Misra P., Shukla D., Mishra S., Srivastava S., Tripathi R.D. (2009). Effect of arsenic on growth, oxidative stress, and antioxidant system in rice seedlings. Ecotoxicol. Environ. Saf..

[18] Tuli R., Chakrabarty D., Trivedi P.K., Tripathi R.D. (2010). Recent advances in arsenic accumulation and metabolism in rice. Mol. Breed..

[19] Srivastava S., D’Souza S.F. (2010). Effect of variable sulfur supply on arsenic tolerance and antioxidant responses in Hydrilla verticillata (L.f.) Royle. Ecotoxicol. Environ. Saf..

[20] Fazili I.S., Jamal A., Ahmad S., Masoodi M., Khan J.S., Abdin M.Z. (2008). Interactive Effect of Sulfur and Nitrogen on Nitrogen Accumulation and Harvest in Oilseed Crops Differing in Nitrogen Assimilation Potential. J. Plant Nutr..

[21] Venegas A., Rigol A., Vidal M. (2015). Viability of organic wastes and biochars as amendments for the remediation of heavy metal-contaminated soils. Chemosphere.

[22] Nelson D.A., Sommers L.E. (1983). Total carbon, organic carbon, and organic matter. Methods Soil Anal..

[23] Murphy J., Riley J.P. (1962). A modified single solution method for the determination of phosphate in natural waters. Anal. Chim. Acta.

[24] Bremner J., Tabatabai M. (1972). Use of an ammonia electrode for determination of ammonium in Kjeldahl analysis of soils. Commun. Soil Sci. Plant Anal..

[25] Chen X.P., Zhu Y.G., Hong M.N., Kappler A., Xu Y.X. (2008). Effects of different forms of nitrogen fertilizers on arsenic uptake by rice plants. Environ. Toxicol. Chem..

[26] Huang L.H., Liu X., Wang Z.C., Liang Z.G., Wang M.M., Liu M., Suarez D.L. (2017). Interactive effects of pH, EC and nitrogen on yields and nutrient absorption of rice (*Oryza sativa* L.). Agric. Water Manag..

[27] Lou Y.L., Zhang Y.S., Lin X.Y. (2005). Effects of f orms of nitrogen fertilizer on the bioavailability of heavy metals in the soils amended with biosolids and their uptake by corn plant. J. Zhejiang Univ..

[28] Chen J., Wang X.J., Zhu L.J. (2004). Effect of pH on adsorption and transformation of arsenic in red soil in GuiZhou. Soils.

[29] Hassan M.J., Wang F., Ali S., Zhang G. (2005). Toxic Effect of Cadmium on Rice as Affected by Nitrogen Fertilizer Form. Plant. Soil..

[30] Tu S., Ma L.Q. (2003). Interactive effects of pH, arsenic and phosphorus on uptake of As and P and growth of the arsenic hyperaccumulator *Pteris vittata* L. under hydroponic conditions. Environ. Exp. Bot..

[31] Yamaguchi N., Nakamura T., Dong D., Takahashi Y., Amachi S., Makino T. (2011). Arsenic release from flooded paddy soils is influenced by speciation, Eh, pH, and iron dissolution. Chemosphere.

[32] Chen H., Shan H.J., Peng S.X., Huang J., Liao D.X., Yan Z.W. (2021). Hydrochemical influences on arsenic adsorption by river sand. Acta Sci. Circumst..

[33] Hu Z.Y., Zhu Y.G., Li M., Zhang L.G., Cao Z.H., Smith F.A. (2007). Sulfur (S)-induced enhancement of iron plaque formation in the rhizosphere reduces arsenic accumulation in rice (*Oryza sativa* L.) seedlings. Environ. Pollut..

[34] Liao X.Y., Chen T.B., Xiao X.Y., Xie H., Yan X.L., Zhai L.M., Wu B. (2007). Selecting appropriate forms of nitrogen fertilizer to enhance soil arsenic removal by Pteris vittata: A new approach in phytoremediation. Int. J. Phytorem..

[35] Xue P.Y., Liu W.J., Liu H.L., Duan G.L., Hu Y. (2010). Arsenic behaviors in the system of arsenic contaminated soil-rhizosphere-rice plants. Acta Pedol. Sin..

[36] Heikens A., Panaullah G.M., Meharg A.A. (2007). Arsenic behaviour from groundwater and soil to crops: Impacts on agriculture and food safety. Rev. Environ. Contam. Toxicol..

[37] Raddatz N., Morales de los Ríos L., Lindahl M., Quintero F.J., Pardo J.M. (2020). Coordinated transport of nitrate, potassium, and sodium. Front. Plant Sci..

[38] Leao G.A., Oliveira J.A., Farnese F.S., Gusman G.S., Felipe R.T. (2014). Sulfur metabolism: Different tolerances of two aquatic macrophytes exposed to arsenic. Ecotoxicol. Environ. Saf..

[39] Nishida S., Duan G.L., Ohkama-Ohtsu N., Uraguchi S., Fujiwara T. (2016). Enhanced arsenic sensitivity with excess phytochelatin accumulation in shoots of a SULTR1;2 knockout mutant of *Arabidopsis thaliana* (L.) Heynh. Soil Sci. Plant Nutr..

[40] National Health and Family Planning Commission of the People’s Republic of China & China Food and Drug Administration (2017). National Food Safety Standard. Maximum Levels of Contaminants in Foods GB2762–2017. https://sppt.cfsa.net.cn:8086/db.

[41] Zhang L., Wang S.R., Jiao L.X., Zhao H.C., Zhang Y., Li Y.P. (2013). Physiological response of a submerged plant (*Myriophyllum spicatum*) to different NH4Cl concentrations in sediments. Ecol. Eng..

[42] Zhou J.M., Dang Z., Chen N.C. (2007). TOC and heavy metals dynamic in contaminated soil solution and their correlations with the addition of chelating agents. Environ. Chem..

