# Supplementary figures and images for: Migration and Transformation of Arsenic in Rice and Soil under Different Nitrogen Sources in Polymetallic Sulfide Mining Areas
Source: Life (Basel). 2022 Oct 4;12(10):1541. doi: 10.3390/life12101541 (PMC9604899; doi:10.3390/life12101541)

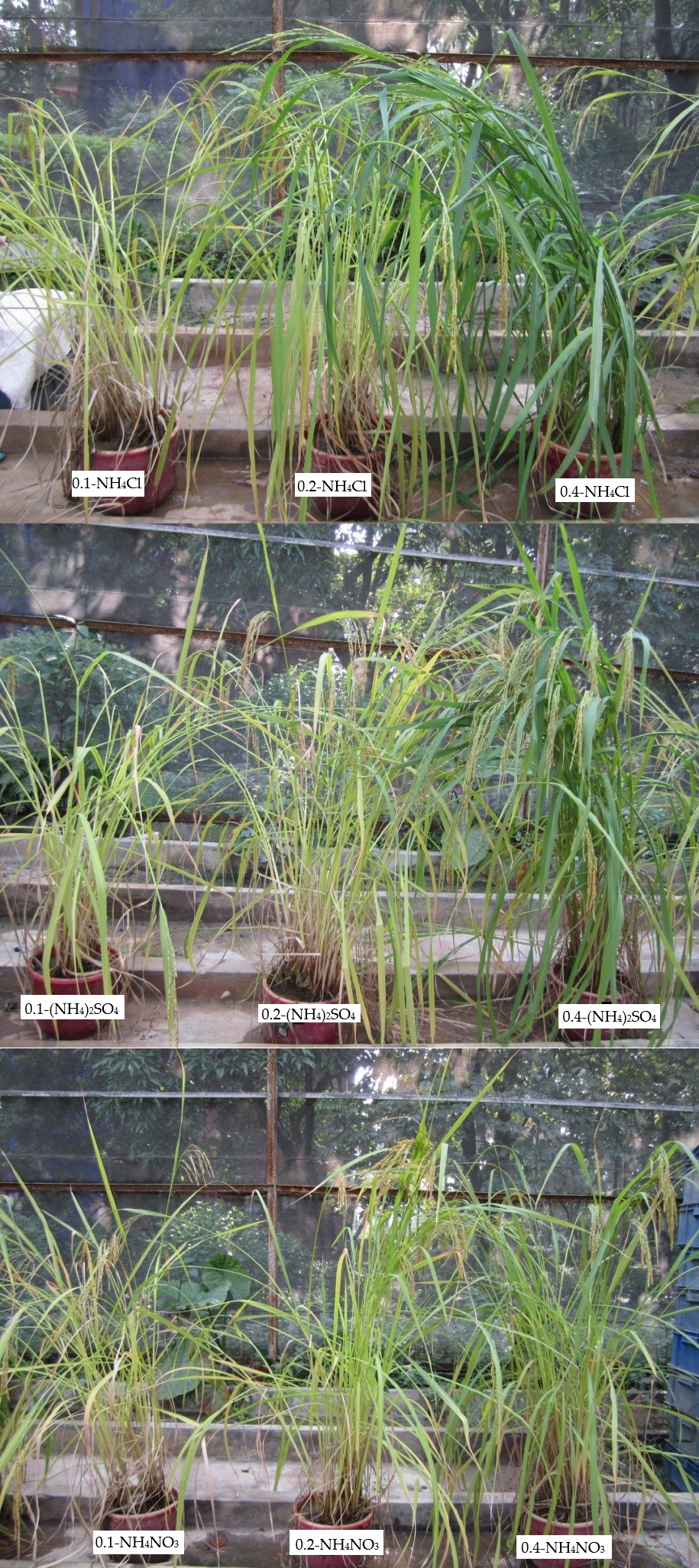

Supplement: Supplementary file 1 [file life-12-01541-s001.zip › life-1902534-supplementary/Figure S1.jpg]
